# Acute spontaneous hematoma of the corpus callosum in a COVID-19 patient: a case report

**DOI:** 10.11604/pamj.2021.38.263.28048

**Published:** 2021-03-15

**Authors:** Boubaker Charra, Ayman Ellouadghiri, Touda Kebbou, Omar Ettouki, Naima El Benna, Moulay Hicham Afif, Mohamed Benghanem Gharbi

**Affiliations:** 1 Department of Anesthesiology and Intensive Care, Ibn Rochd University Hospital of Casablanca, Hassan II University, Casablanca, Morocco,; 2 Department of Radiology, Ibn Rochd University Hospital of Casablanca, Hassan II University, Casablanca, Morocco,; 3 Department of Pneumology, Ibn Rochd University Hospital of Casablanca, Hassan II University, Casablanca, Morocco,; 4 Department of Nephrology, Ibn Rochd University Hospital of Casablanca, Hassan II University, Casablanca, Morocco

**Keywords:** COVID-19, corpus callosum, hematoma, case report

## Abstract

Neurovascular involvement is a frequent occurring reported in COVID-19 patients. However, spontaneous hematomas of the corpus callosum are exceptionally seen. The authors of this article aim to report an unusual case of corpus callosum hematoma in a COVID-19 patient and discuss potential etiologies and mechanisms responsible for intracranial hemorrhage.

## Introduction

SARS-CoV-2 has been known to be responsible of various symptoms. Pneumonia is the most typical manifestation; however, neurological, vascular and gastrointestinal involvement have been previously reported. Ischemic strokes due to COVID-19 are frequent, however intracranial hemorrhage is less likely to happen [1]. The authors of this article report a rare case of association of COVID-19 infection and corpus callosum hematoma.

## Patient and observation

A 61-year-old male patient with history of recurrent thrombophlebitis was admitted to a local hospital for dyspnea, fever and dry cough. A nasopharyngeal sample was tested positive via RT-PCR for SARS-CoV-2. The patient received treatment and was monitored for a week before presenting psychomotor agitation and aggravating his respiratory symptoms. He was then intubated and transferred to the intensive care unit for further investigations and monitoring. First examination revealed a sedated, intubated patient, with protective ventilation parameters. His hemodynamic status was stable. He didn´t present any focal neurological deficit and his pupils were equal and reactive. Temperature was of 38°C. Electrocardiograph (EKG) showed a sinusal tachycardia of 141 bpm. A non-contrast chest computed tomography (CT) was conducted and showed bilateral diffuse ground glass opacities covering more than 75% of the parenchyma. Echocardiography revealed altered left ventricular contractility, elevated left ventricular filling pressures and E/E´ = 11, with a dilated right ventricle. RV/LV ratio of 1. Arterial gasometry revealed respiratory acidosis. Pertinent laboratory values included a lymphopenia of 660 el/mm^3^, neutrophil (12400 el/mm^3^) predominant hyperleukocytosis of 13840 el/mm^3^, and normal platelet count of 306000 el/mm^3^. Hemostasis levels were of PT 24%, pTT of 40sec and fibrinogen of 6g/L. D-dimers of 1500ng/mL. Hepatic function was altered with elevated with ALT of 942IU/L and spartate aminotransferase (AST) of 1299IU/L. Renal function levels were mildly elevated with blood creatinine of 19mg/L and urea of 0.62g/L. Electrolyte blood panel was normal.

**Figure 1:**
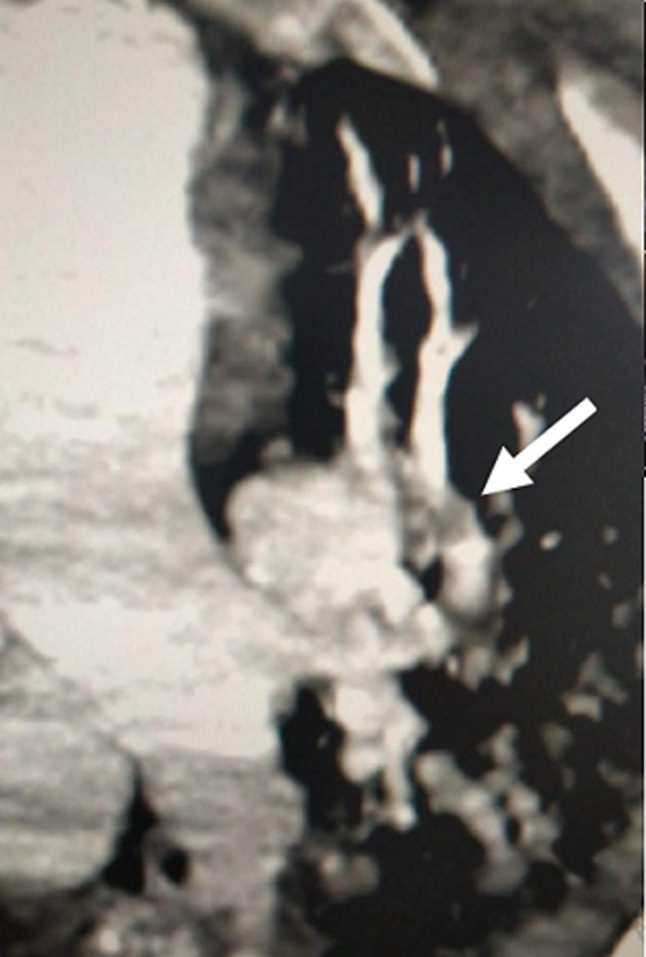
subocclusive pulmonary embolism (white arrow)

**Figure 2:**
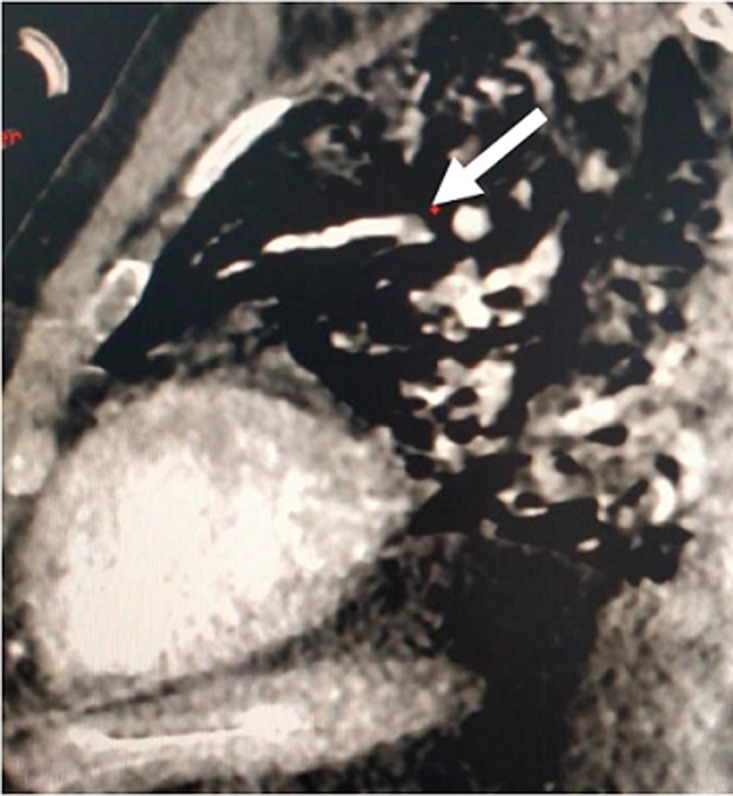
subocclusive pulmonary embolism, sagittal view (white arrow)

**Figure 3:**
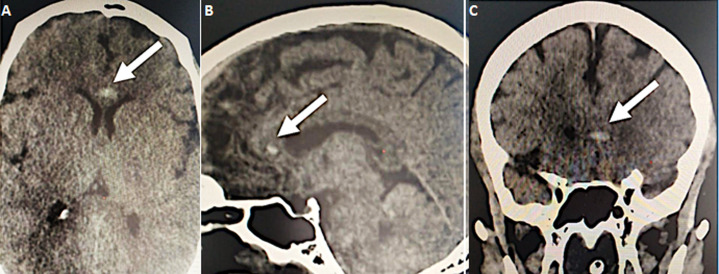
A) hematoma of the corpus callosum rostrum, axial view (white arrow); B) hematoma of the corpus callosum rostrum, sagittal view (white arrow); C) subocclusive pulmonary embolism, sagittal view (white arrow)

Further investigations were conducted to explore hepatic dysfunction: negative hepatitis serologies and autoimmune tests. Blood paracetamol level was normal. Factor V level was normal of 89%. Our therapeutic care was based on Ceftriaxon (2g per day) and Moxifloxacin (400 mg, twice a day), proton pump inhibitors, vitamin C, Vitamin D and Zinc. The patient received fresh frozen plasma transfusion, N-acetyl-cystein which led to a stabilization of his hemostasis levels. Preventive anti-coagulation was introduced in the treatment. The evolution was marked with a worsening of his respiratory status and renal function. Hemodialysis was indicated and realized. Venous doppler of lower limbs was conducted and revealed left deep venous thrombosis. Echocardiography showed right ventricle dysfunction as a sign of acute pulmonary embolism which was confirmed with CT angiogram (Figure 1, Figure 2). Curative doses of unfractionned heparin were introduced. Sedation was reduced after two weeks; however, the patient did not regain consciousness. Cerebral CT was performed and revealed a spontaneous corpus callosum rostrum hematoma (Figure 3).

## Discussion

COVID-19 has been associated with a wide range of symptoms including hematologic, vascular and neurological [2]. The most frequent neurovascular findings are acute ischemic strokes, intracranial hemorrhage including microhemorrhage and thromboembolic episodes. Our patient presented a spontaneous hematoma of the corpus callosum rostrum. The involvement of this organ has been previously described, as ischemic, cytotoxic lesions predominantly in the posterior corpus callosum [3]. It is mainly due to cytokine storm, and hypoxemia-induced high concentrations of glutamate [4]. However, another study has shown that intracranial hemorrhage in COVID-19 patients is mostly considered as hemorrhagic transformation of ischemic infarct rather than spontaneous hematoma [5]. Subsequent hemorrhage is most likely multifactorial [6]. As patients who showed intracranial hemorrhage usually have other risk factors. Our patient had history of thrombophlebitis seven years prior to his pneumonia, a recurrent thrombophlebitis with pulmonary embolism during his stay, as well as altered hemostasis. Cerebral CT did not show other signs of ischemia, and deep thrombophlebitis cannot be excluded as he could not have magnetic resonance (MR)-angiography. Anticoagulation, also used for our patient, may predispose to hemorrhage. Additionally, he underwent hemodialysis, worsening his hematological status and increasing intracranial hemorrhage risk [7,8]. Previous studies have incriminated angiotensin-converting enzyme II (ACE II) receptors, present in cerebrovascular endothelial cells. SARS-CoV-2 has been showed to use these receptors as a means of entry inside cerebral cells [9]. Thus, a dysfunction of ACE II receptors may cause disruption of autoregulation as well as blood pressure spikes due to arterial wall rupture, leading to intracranial hemorrhage [10].

## Conclusion

COVID-19 is a multisystemic disease with a panoply of abnormalities. Corpus callosum involvement is known to be ischemic, however this case of spontaneous hematoma may be a result of many predisposing factors starting from recurrent thrombophlebitis, hematologic dysfunction to hemodialysis. Intracranial hemorrhage may in this case, not only be a consequence of hemostasis alteration but subsequent to the virus itself.
